# Transcriptome analysis to identify the downstream genes of androgen receptor in dermal papilla cells

**DOI:** 10.1186/s12863-021-01018-6

**Published:** 2022-01-04

**Authors:** Kai Furuya, So Fujibayashi, Tao Wu, Kouhei Takahashi, Shin Takase, Ai Orimoto, Eriko Sugano, Hiroshi Tomita, Sayo Kashiwagi, Tohru Kiyono, Tsuyoshi Ishii, Tomokazu Fukuda

**Affiliations:** 1 Graduate School of Science and Engineering, Iwate University, 4-3-5 Ueda, Morioka, Iwate 020-8551 Japan; 2 Rohto Pharmaceutical Co., Ltd., Basic Research Development Division, 6-5-4 Kunimidai, Kizugawa, Kyoto 619–0216 Japan; 3 Exploratory Oncology Research and Clinical Trial Center, National Cancer Center, 6-5-1 Kashiwanoha, Kashiwa, Chiba 277–8577 Japan

**Keywords:** Androgen receptor, Dermal papilla cells, RNA-Seq

## Abstract

**Background:**

Testosterone signaling mediates various diseases, such as androgenetic alopecia and prostate cancer. Testosterone signaling is mediated by the androgen receptor (AR). In this study, we fortuitously found that primary and immortalized dermal papilla cells suppressed AR expression, although dermal papilla cells express AR in vivo. To analyze the AR signaling pathway, we exogenously introduced the AR gene via a retrovirus into immortalized dermal papilla cells and comprehensively compared their expression profiles with and without AR expression.

**Results:**

Whole-transcriptome profiling revealed that the focal adhesion pathway was mainly affected by the activation of AR signaling. In particular, we found that caveolin-1 gene expression was downregulated in AR-expressing cells, suggesting that caveolin-1 is controlled by AR.

**Conclusion:**

Our whole transcriptome data is critical resources for discovery of new therapeutic targets for testosterone-related diseases.

**Supplementary Information:**

The online version contains supplementary material available at 10.1186/s12863-021-01018-6.

## Highlight

The comprehensive gene expression profiling were obtained by RNA-Seq analysis about AR negative and AR positive dermal papilla cells.

The bioinformatics analysis suggested that caveolin-1 and EGF receptors are the downstream of AR signaling.

Our study showed the combination of pinpoint mutant cells and global transcriptome is effective to identify the downstream genes.

**Supplementary Information:**

The online version contains supplementary material available at 10.1186/s12863-021-01018-6.

## Code availability

The software and its versions were used for the data analysis.

FastQC, version 0.11.8 was used for quality check of raw FASTQ sequencing file. https://www.bioinformatics.babraham.ac.uk/projects/fastqc/

PRINSEQ, version 0.20.4 was used for the removal of low quality reads. http://prinseq.sourceforge.net/

PEAT, version 1.2 was used for the removal of the adaptor sequence. https://github.com/jhhung/PEAT

STAR, version 2.6.1 was used for the mapping. https://github.com/alexdobin/STAR

featureCount, SUBREAD, release 1.6.5 was used for the expression counting. http://subread.sourceforge.net

R package, version 4.0.3, was used for the downstream analysis. https://www.r-project.org

TCC-GUI, tool for the downstream analysis. https://github.com/swsoyee/TCC-GUI

## Background

Testosterone is a hormone that controls cell growth or sexual differentiation of the reproductive organs. This hormone is known to be associated with various diseases, such as androgenetic alopecia (AGA) and prostate cancer. Approximately 30% of males are reported to be affected by AGA [1]. The mechanism of AGA progression is the activation of AR signaling and secretion of Dickkopf-related protein 1 (DKK1) or tumor growth factor-β2 (TGF-β2), which suppress the growth of hair matrix cells [2]. Testosterone signaling is mainly mediated by the androgen receptor (AR) [3]. The activation of AR signaling starts with the binding of ligands, such as testosterone or dihydroxy-testosterone, which has a high affinity for AR. Ligand-activated AR forms a dimer on the cellular membrane and translocates from the cytoplasm to the nucleus. After nuclear translocation, AR forms a transcriptional complex, including coactivators and RNA polymerase, resulting in the transcriptional activation of downstream genes [3]. In prostate cancer, the growth of cancer cells strongly depends on the activation of testosterone [4]. Therefore, the identification of a chemical that can inhibit testosterone signaling may provide a strong candidate for the treatment of prostate cancer and/or AGA.

The hair growth is controlled by the growth signal from human follicle dermal papilla cells (HFDPCs). Interestingly, Kwack et al. showed that primary HFDPCs cause a decrease in AR expression even after passage 6. In agreement with this phenomenon, we could not measure the expression level of AR, even in early passages of primary HFDPCs [5]. Although the detailed mechanism is not clear, the expression level of AR dramatically decreases after sequential passages of the DPCs. The immortalized cells established from corresponding primary cells were also negative for AR expression [5]. These situations led us to hypothesize that we could reconstitute the AR signaling pathway if AR is exogenously introduced. In fact, we introduced an expression cassette of AR with a hemagglutinin (HA) tag using a retrovirus in our previous study [5]. Interestingly, the introduction of exogenous AR caused elevated expression of DKK1, indicating that the AR signaling pathway remains intact in HFDPCs even after immortalization [5]. Therefore, we concluded that we successfully obtained genetic mutants of HFDPCs that were negative or positive for AR expression. The comparison of expression profiles between AR-positive and AR-negative immortalized DPCs would allow us to identify the downstream genes of AR. In this study, we comprehensively compared the expression profiles of immortalized HFDPCs with or without AR expression to identify the downstream gene network of AR signaling, which will contribute to finding out of new target molecules to androgen related diseases.

## Methods

### Cell culture and RNA extraction

We previously reported the establishment of immortalized DPCs with the expression of R24C mutant cyclin-dependent kinase 4 (CDK4), cyclin D1, and telomerase reverse transcriptase (TERT) via lentiviral gene transfer. The immortalized human follicle dermal papilla cells were named as “HFDPC_K4DT” from the last characters of the introduced genes (CDK4, cyclin D1, TERT). We previously confirmed that AR expression in established immortalized cells (HFDPC_K4DT) is undetectable, almost identical to that of fibroblasts. To reconstitute the AR signaling pathway, we introduced an expression cassette of AR with an HA tag through the retrovirus expression system. Total RNA was extracted from immortalized HFDPCs using the K4DT method and AR-expressing immortalized HFDPCs. To detect the reproducibility of the expression counts, we extracted RNA from three biological replicates.

### RNA-Seq analysis

We checked the quality of the raw sequencing reads using FastQC. Data were obtained using paired-end sequencing. We analyzed the data from six samples of HFDPC-K4DT and AR-expressing HFDPC-K4DT. Raw sequencing reads were processed with PEAT program to remove the adaptor sequence. We removed the low-quality reads using PRINSEQ, and the remaining reads were mapped to the human reference genome (GRC38, NCBI) with the STAR mapping program. The expression counts for each sample were obtained using the featureCounts program. We first extracted genes which have more than 3000 counts on any sample. To normalize expression counts, we used the DEGES normalization method [6]. We set the parameters of TCC-GUI as default setting [7, 8] The differentially expressed genes (DEGs) were determined using the TCC-GUI program developed by Dr. Koji Kadota (Tokyo University, Tokyo, Japan; https://infinityloop.shinyapps.io/TCC-GUI/). For the determination of differentially expressed genes, we used Q value (FDR) less than 0.01.

### Downstream pathway analysis

After mapping all sequencing reads, the expression count of all genes was determined (33,122 genes). Based on the results of DEGs in TCC-GUI, we extracted 1196 genes classified as DEGs. The list of DEGs was processed using the DAVID pathway analysis tool. The expression levels of genes in the listed pathways were visualized using the heatmap function of TCC-GUI.

### Detection of expression level of caveolin-1 and EGFR with qPCR

The total RNAs of HFDPC-K4DT and AR expressing K4DT cells were extracted with NucleoSpin RNA (Takara Bio, Shiga, Japan). The cells were treated with basal medium containing 50 nM DHT or without DHT (no treatement) for 8 h. Total cDNAs were obatained with PrimeScript RT reagent Kit with gDNA Eraser with random primer method. The cDNA were used as the template samples of qRT-PCR with THUNDERBIRD SYBR qPCR Mix (TOYOBO, Osaka, Japan) under the reaction condition recommended from the manufacture. The sequences of the detection primers were listed in below. Primers for caveolin 1, TF1162; 5′-CCCGCAGCCTGGGAGTGCCCTGA-3′ and TF1163, 5′-GCTTGTAGATGTTGCCCTGTTCCCGGAT-3′. Primers for EGFP, TF1164, 5′-ATGATGCAAATAAAACCGGACTGAAGGA-3′, TF1165, 5′-CTGCACCCCAGCAGCTCCCATTG-3′. Primers for GAPDH, TF999, 5′- GAGGTGCACCACCAACTGCTTAGC-3′ and TF1000, 5′-TCGGCATGGACTGTGGTCATGAG-3′. The detections with qRT-PCR were carried out with Thermal Cycler Dice Real Time System II (Takara Bio, Shiga, Japan) under the relative quantitation with GAPDH.

## Results

### Biological background of immortalized HFDPCs and AR-expressing immortalized cells

**Fig. 1:**
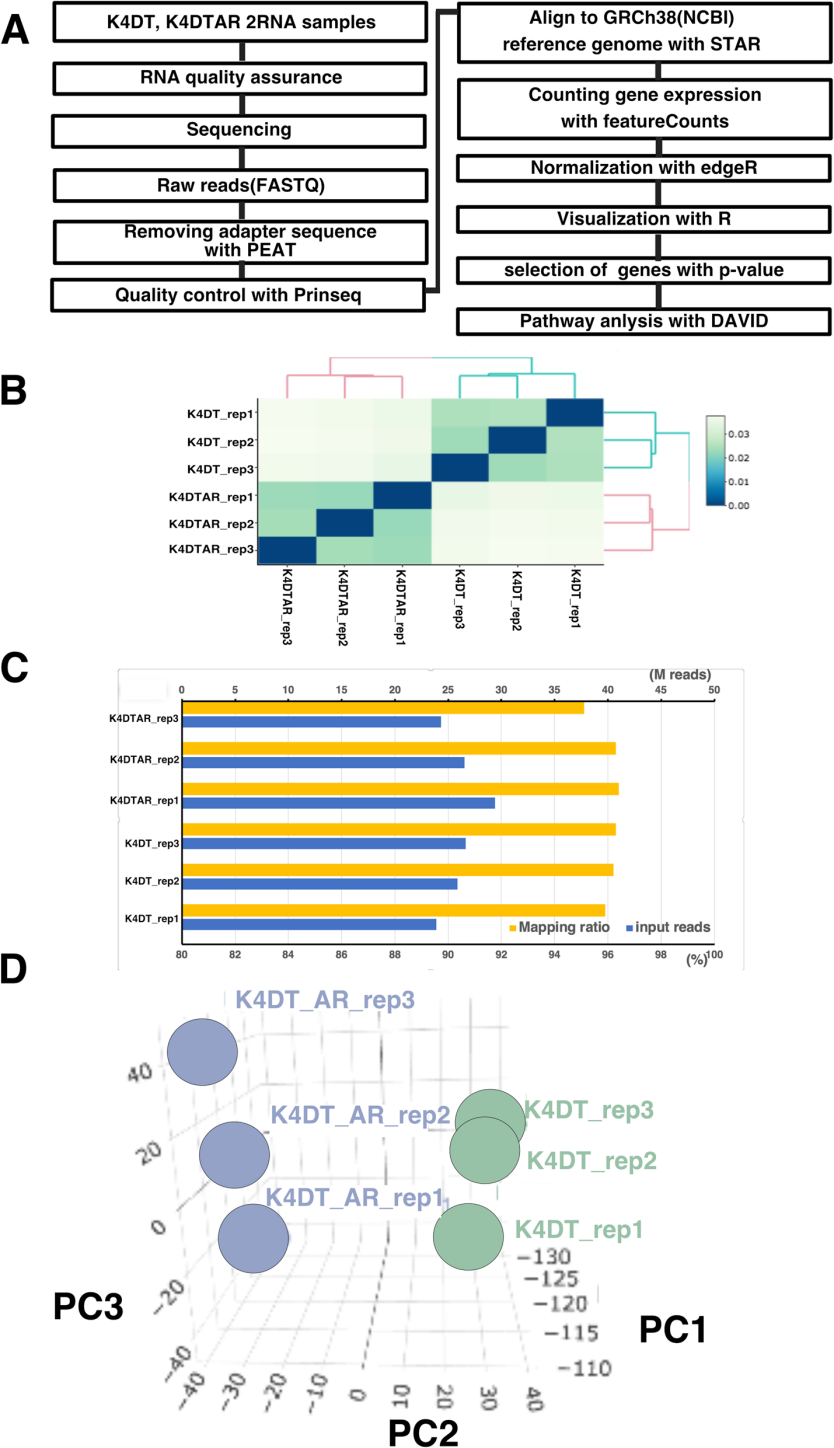
Workflow of RNA-Seq analysis and PCA of immortalized human dermal papilla cells (HFDPCs) with K4DT cells and AR-expressing cells. **A** Workflow of the analysis. **B** Correlation matrix of all samples. Triplicate samples formed unique clusters. **C** Mapping ratio of parent immortalized HFDPC-K4DT cells and AR-expressing HFDPC-K4DT cells. The mapping results of parent K4DT were reproduced from our previous publication (Fukuda T., et al., 24: 101929, iScience, 2012). **D** PCA of expression profiling of HFDPC-K4DT cells with and without AR expression

**Fig. 2:**
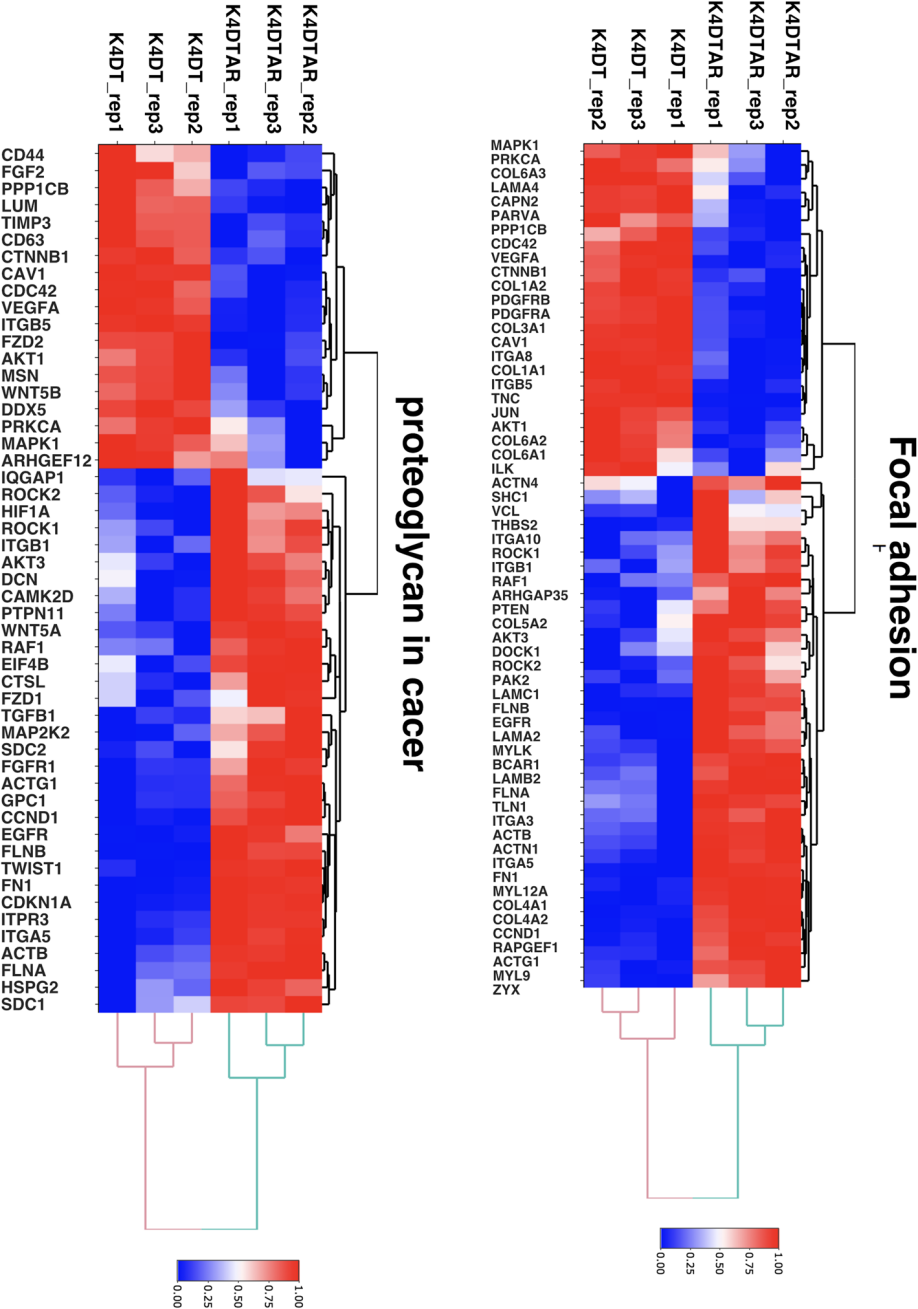
Heatmap analysis of differentially expressed genes listed in focal adhesion pathway and Proteoglycans in cancer pathway, which are listed in the KEGG database. Red indicates high expression and blue indicates low expression of genes

In our previous study, we exogenously introduced an AR expression cassette through retrovirus gene transfer. Although the expression level of AR was undetectable in parent cells, the AR-expressing immortalized HFDPCs showed an intense signal with the expected molecular weight in western blotting (see Fig. 1 of our previous publication, Fukuda et al., 2020). Furthermore, we detected the nuclear localization of AR even without ligand stimulation, which may explain auto-dimerization based on the force expression system (see Fig. 2 of our previous publication, in Fukuda et al., 2020). Furthermore, we detected activation of DKK1 (a major downstream gene of AR) expression in AR-expressing immortalized HFDPCs, which indicated that the AR signaling pathway was reconstituted by exogenous AR introduction.

### Whole-gene transcriptome analysis of parent HFDPC-K4DT and AR-expressing HFDPC-K4DT

To comprehensively compare the expression patterns of whole genes, we carried out RNA-Seq analysis using an Illumina Hiseq X sequencing machine (Illumina, San Diego, CA, USA) and a 150-bp paired end. The sequencing workflow is shown in Fig. 1A. After the removal of the adaptor, we initiated the mapping process. The number of obtained sequence reads was at least 22 M, indicating that the read number was sufficient for quantitation [9]. To evaluate the reproducibility of the data, we carried out RNA-Seq reactions with three biological replicates. We evaluated the quality of the read data using the FASTQC program (Fig. S1 and S2). The results of FASTQC indicated that the average of almost all sequencing data was mapped within the green area, suggesting that the read data was reliable. We next mapped sequencing reads using the STAR program and human reference genome (GRCh38). The mapping ratio and read number are shown in Fig. 1C. The mapping ratio of the samples was more than 95%, indicating that our mapping method was suitable for detecting gene expression. The mapping ratios were 95.9% (HFDPC_K4DT1), 96.2% (HFDPC_K4DT2), 96.3% (HFDPC_K4DT3), 96.4% (HFDPC_K4DT_AR1), 96.3% (HFDPC_K4DT_AR2), and 95.1% (HFDPC_K4DT_AR3).

**Fig. 3:**
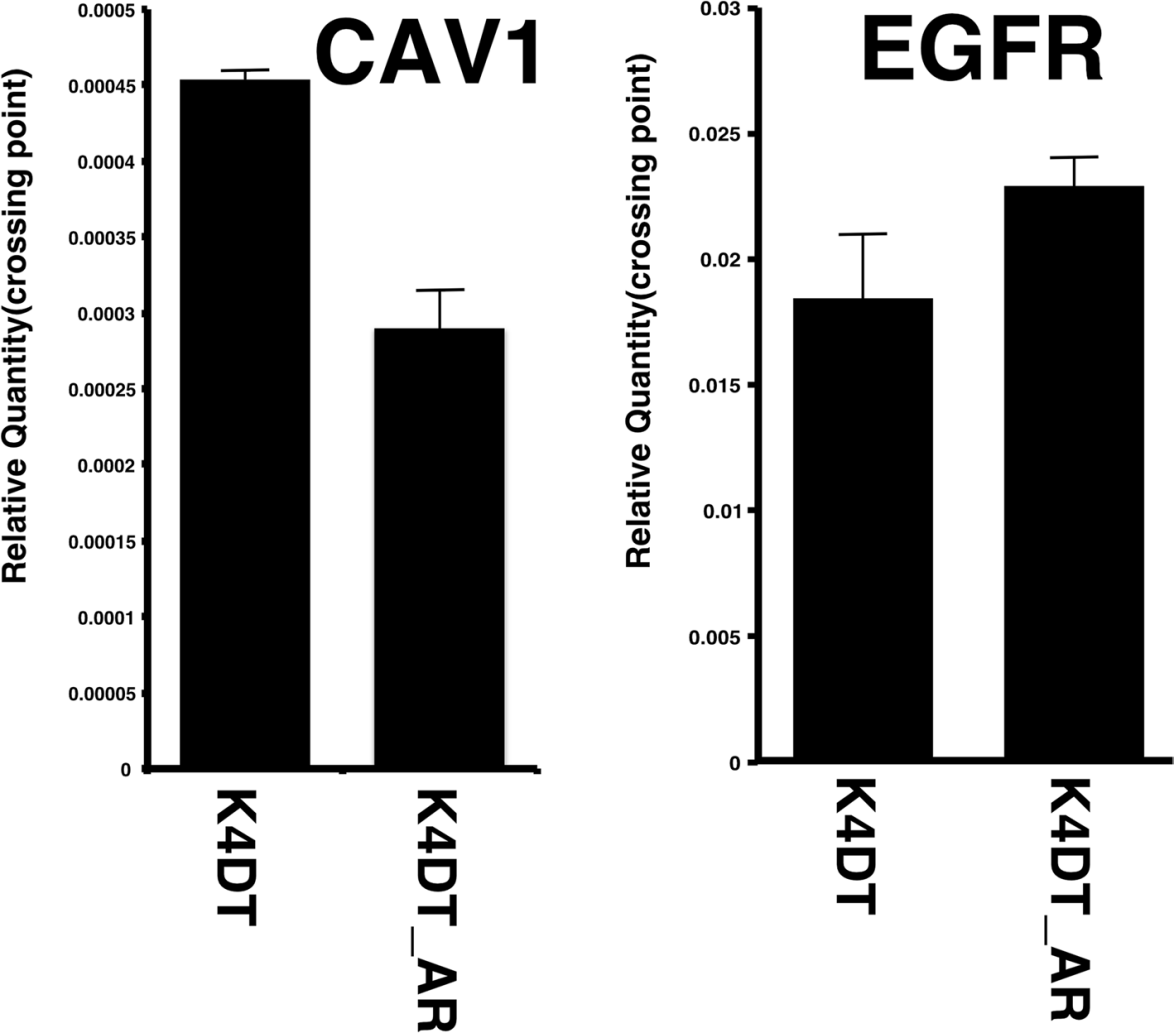
Expression level of caveolin-1 and EGF receptors detected by qRT-PCR in identical RNAs, which used for RNA-Seq. Relative RNA quantality which detected with internal control (GAPDH) were shown in the graph. Average value and standard errors are shown with biological triplicated samples

The complete list of expression counts of parent HFDPC-K4DT and AR-expressing HFDPC-K4DT is provided in Figshare (https://figshare.com/articles/dataset/HFDPC_K4DT_HFDPC_K4DT_AR/13567343). The sequencing data were submitted to the DDBJ database under Bioproject **Submission ID PRJDB10909.** We first filtered genes at least 3000 counts on any sample. The number of genes remained after the filtration was 1537 genes. Next, we input the expression counts of the whole genome into TCC-GUI. First, we analyzed the correlation plots of expression profiles, as shown in Fig. 1B. The biological replicates of parent HFDPC-K4DT and AR-expressing HFDPC-K4DT formed unique clusters, indicating that the sequencing results were reproducible. The triplicated data also formed unique clusters in three-dimensional PCA (Fig. 1).

### Pathway analysis results

We further analyzed the DEGs with a FDR based Q value of less than 0.01. In total, we narrowed down the DEGs to 1196 as candidate genes. The list of DEGs was submitted to the pathway analysis tool DAVID. The most significant pathway determined in DAVID was the focal adhesion pathway (61 hits in the annotation list), followed by Proteoglycans in cancer (51 hits in the annotation list). Based on the pathway analysis results, we compared 61 genes listed in focal adhesion using heatmap analysis (Fig. 2). Furthermore, the expression levels of Proteoglycans in cancer related genes (51 genes) are shown in Fig. 6. We also mapped the DEGs in the Kyoto Encyclopedia of Genes and Genomes (KEGG), which showed more than a 2-fold increase or 0.5-fold decrease (at least 3000 counts in any sample) based on bar plots of expression counts and decision criteria [10–12]. Mapping of the focal adhesion pathway indicated that collagen-related molecules (FN1, COL1A1, COL27A1, COL4A1, COL4A2, and COL5A3) were either downregulated or upregulated in AR-expressing cells. Furthermore, the expression level of Caveolin 1 was downregulated and EGFR was upregulated in AR expressing HFDPC-K4DT cell (Fig. 5).

**Fig. 4:**
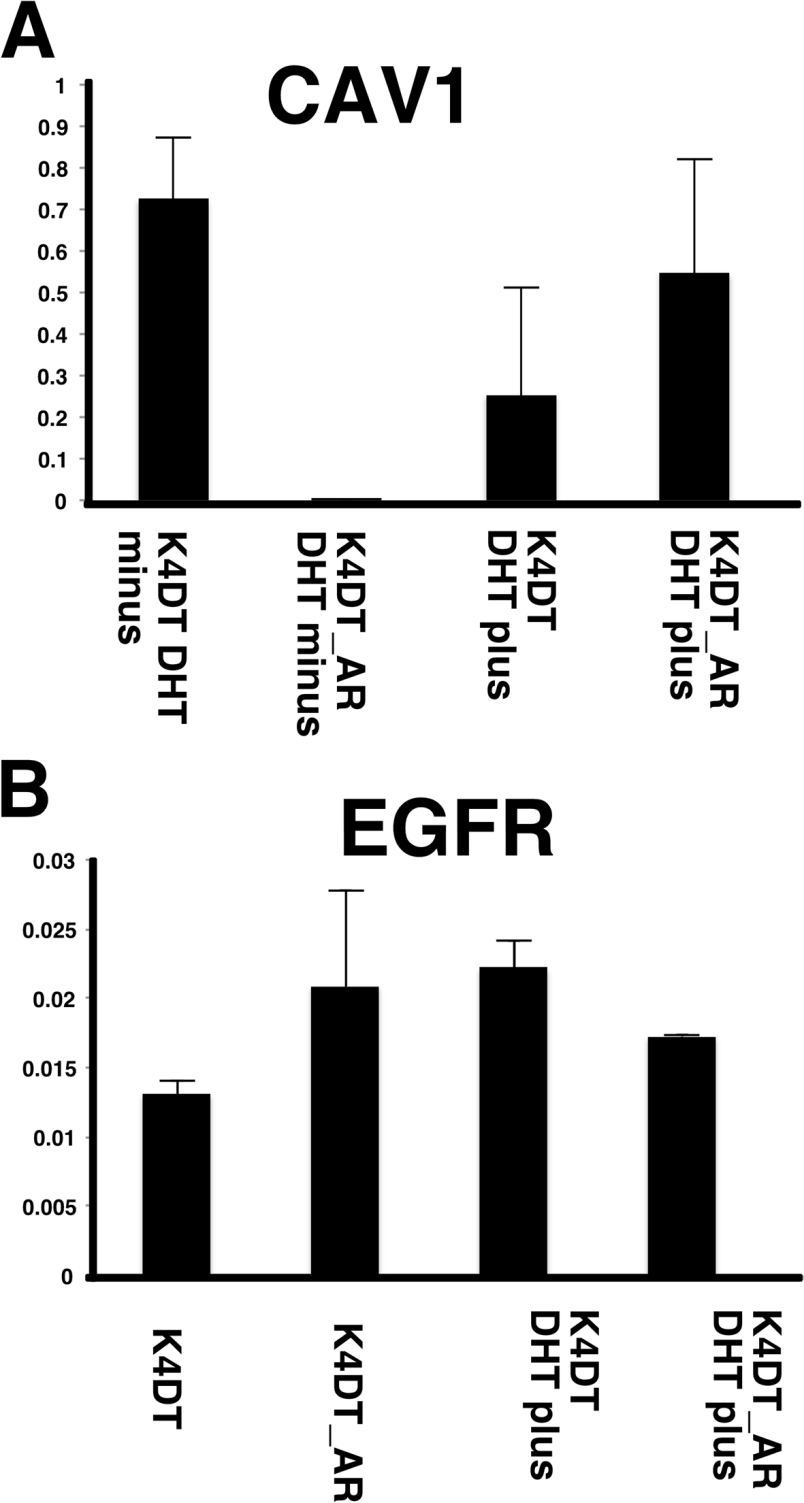
Expression level of caveolin-1 and EGF receptors in HFDPC-K4DT parental cell, and AR expressing HFDPC-K4DT cell, and after the treatment of 50 nM of DHT treatment. **A** Expression level of caveolin-1, before treatment of DHT (Left side), and after the 8 h of DHT treatment (Right side). **B** Expression level of EGFR, before before treatment of DHT (Left side), and after the 8 h of DHT treatment (Right side). Average value and standard error are shown with biological triplicated samples

In Proteoglycans in cancer related pathway, the expression level of Twist was elevated which is one of the transcriptional factor in AR expressing cell.

### Validation of RNA-Seq results with qPCR analysis

We furthermore detected the expression levels of Caveolin 1 downregulated and EGFR with qPCR analysis. As the first evidence, we detected expression of these two genes in identical RNAs which used for RNA-Seq. The downregulation of Caveolin 1 and upregulation of EGFR in AR expressing cell were reproduced with qPCR analysis (Fig. 3). Furthermore, we carried out treatment of 50 nM of dihydrotestosterone (DHT) with and without AR expressing cells. Under the intact condition, downregulation of Caveolin 1 and upregulation of EGFR in AR expressing cell were also reproduced (Fig. 4A and B, Left side). However, expression of Caveolin 1 was strongly suppressed after the treatment of DHT even in parent K4DT cell. The expression levek of Caveolin 1 was elevated in AR expressing cells. These data indicate that 50 nM DHT treatment causes various types of expression change, which independent to AR signaling.

**Fig. 5:**
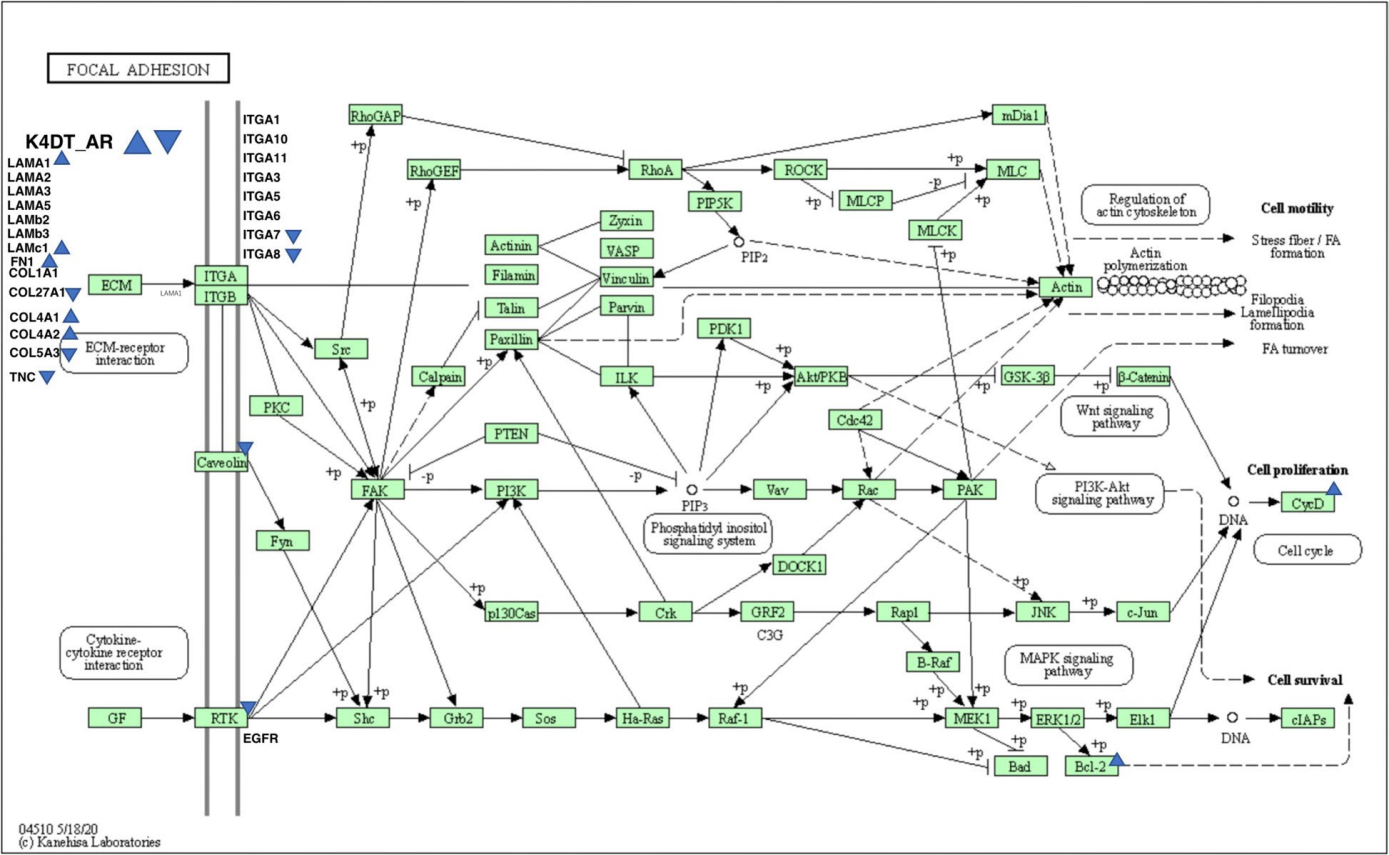
Location of upregulated or downregulated genes in AR-expressing HFDPC-K4DT cells in focal adhesion pathway from KEGG

**Fig. 6:**
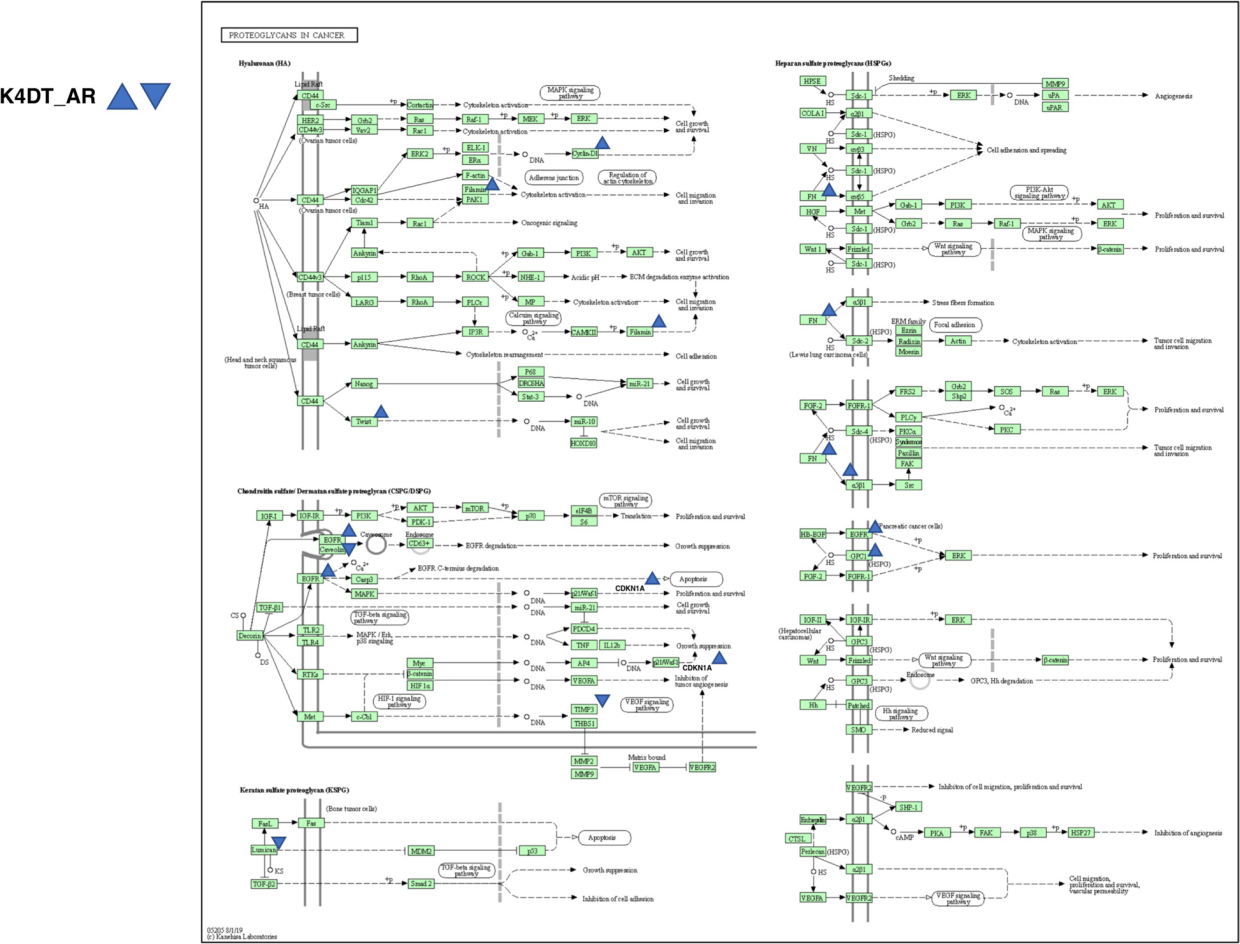
Location of upregulated or downregulated genes in AR-expressing HFDPCK4DT cell in Proteoglycans in cancer pathway from KEGG

## Discussion

In this study, we comprehensively compared the expression profiles of parent immortalized human HFDPCs with K4DT and AR-expressing offspring cells. The genetic background of these cell lines was identical except for the expression status of AR. The comparison of global expression profiles allowed us to identify the downstream genes involved in AR signaling.

We already detected that HA-labelled androgen receptor get into the nuclei without treatment of testosterone in AR expressing cell in our previous publication. The nuclear localization of exogenously introduced HA-labelled androgen receptor without ligand treatment can be possibly explained by following two reasons; first possibility is the auto-dimerization. Due to the high level of expression of HA-labelled AR, receptor might cause auto-dimerization, which is critical process for the activation of AR signaling. The second possibility is the testosterone concentration within the serum in cell culture medium. Since the testosterone is quite sensitive as the hormone receptor, we need to perform serum withdrawn to exactly detect the nuclear translocation. We furthermore carried out the exposure of 50 nM DHT to parent K4DT cell and AR expressing cell. The exposure of DHT causes the elevation of Caveolin 1 even in the AR negative parent cell. The results of DHT exposure in parent cell showed that DHT treatment causes changes in signaling pathway, which independent from AR manner. We need to pay attention for the use of the ligand. If we use synthetic antrogen recptor ligand, such as R1881, the effect of ligand treatment might show different response. However, although the synthetic ligand might be specific from the view point of androgen signaling pathway, the biological explanation would be difficult when it compared with natural ligands such as DHT.

We found that caveolin-1 is downregulated in AR-expressing cells. In support of this result, caveolin-1 is reported to be controlled by AR signaling [13]. Furthermore, caveolin-1 has been identified as a malignant marker of prostate cancer, and it controls the survival ratio of prostate cancer cells [14, 15]. In addition, in a mouse study, caveolin-1 expression was suppressed after testosterone treatment. The expression level of caveolin-1 increased in castrated male mice [13]. In the mouse genome, the two binding sites were identified within intron 2, indicating that caveolin-1 expression is controlled by AR. Although the association of caveolin-1 and AR has been suggested in previous studies, the connection between these two molecules has not yet been fully elucidated. Our expression profiling data suggest that the strength of the AR signaling pathway is controlled by caveolin-1. We also identified the expression level of EGFR is upregulated in AR expressing cell. Identification of EGFR as the downstream suggest the cross-talk of AR and EGF signals. These data indicate the existence of a gene network under the control of the AR nuclear receptor. Improved understanding of AR-related networks may contribute to the discovery of new therapeutic targets for AR-related diseases, such as prostate cancer or AGA.

## Conclusion

Our whole transcriptome data is critical resources for discovery of new therapeutic targets for testosterone-related diseases.

## Supplementary Information

**Additional file 1.**

**Additional file 2.**

**Additional file 3.**

**Additional file 4.**

**Additional file 5.**

**Additional file 6.**

**Additional file 7.**

**Additional file 8.**

**Additional file 9.**

**Additional file 10.**

**Additional file 11.**

## Data Availability

The datasets generated during and/or analysed during the current study are available in the Figshare and DNA data base of Japan (DDBJ repository, [https://figshare.com/articles/dataset/HFDPC_K4DT_HFDPC_K4DT_AR/13567343] and Bioproject Submission ID PRJDB10909, https://www.ncbi.nlm.nih.gov/bioproject/686284.

## Abbreviations

AGA: Androgenetic alopecia
AR: Androgen receptor
CDK4: Cyclin-dependent kinase 4
DKK1: Dickkopf-related protein 1
FDR: False discovery rate
GUI: Graphical user interface
GAPDH: Human follicle dermal papilla cells
HFDPC: Glyceraldehyde-3-phosphate dehydrogenase
HA: Hemagglutinin
PCA: Principle component analysis
TCC: Tag count comparison
TERT: Telomerase reverse transcriptase
TGF-β2: Tumor growth factor-β2
