# Supplementary figures and images for: Transcriptome analysis to identify the downstream genes of androgen receptor in dermal papilla cells
Source: BMC Genom Data. 2022 Jan 4;23:2. doi: 10.1186/s12863-021-01018-6 (PMC8725446; doi:10.1186/s12863-021-01018-6)

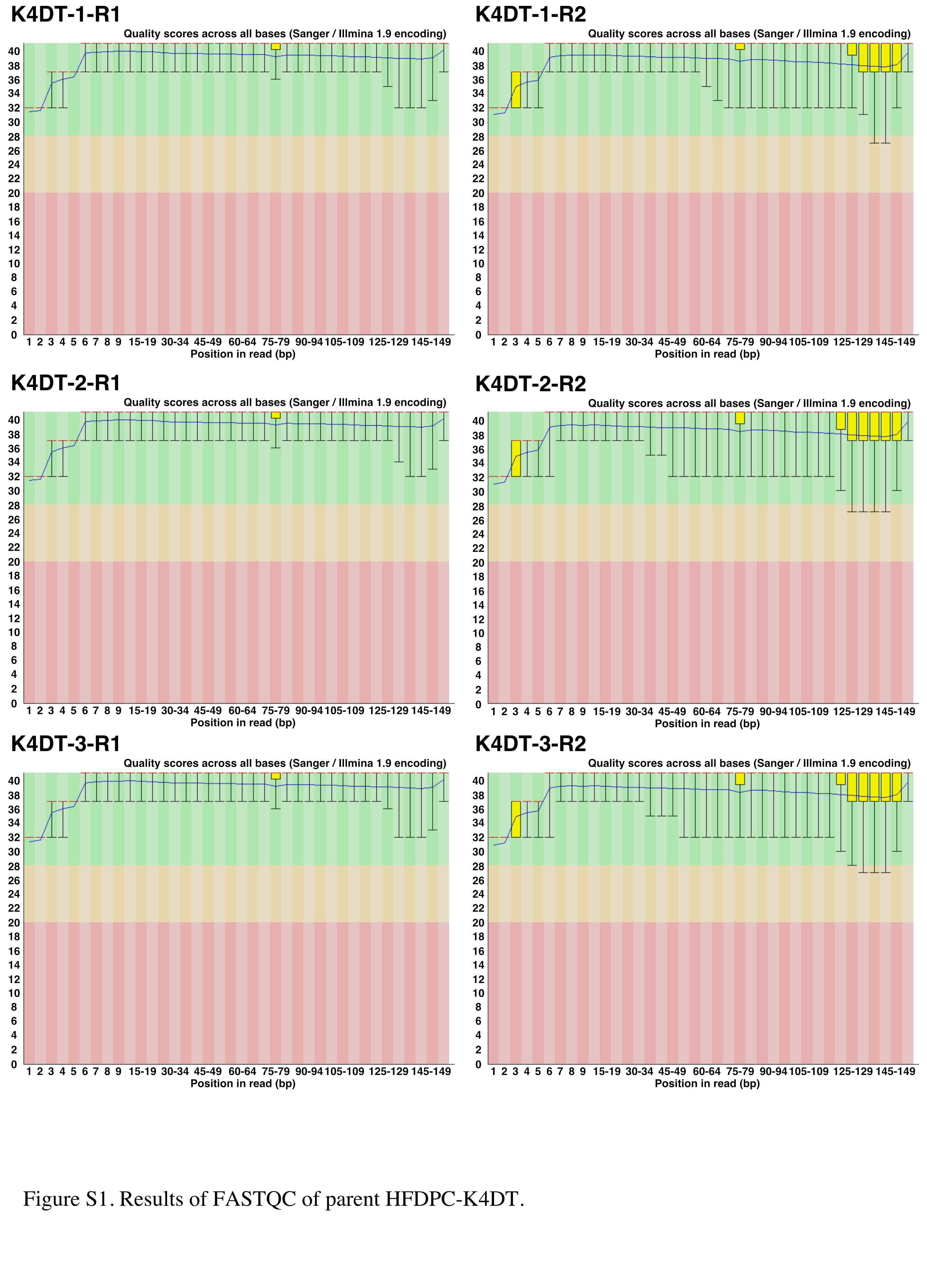

Supplement: Supplementary file 1 — Additional file 1. [file 12863_2021_1018_MOESM1_ESM.tiff]

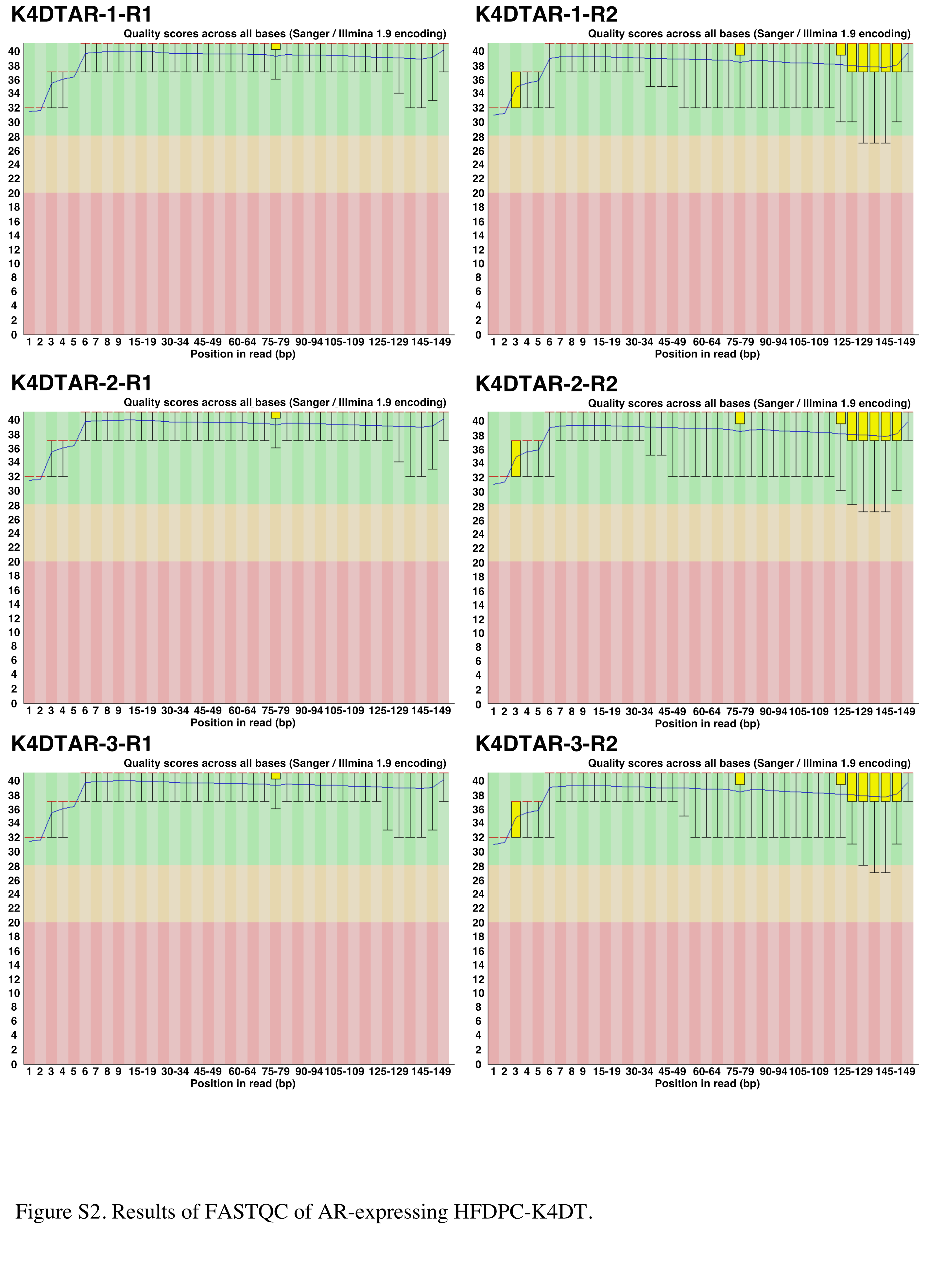

Supplement: Supplementary file 2 — Additional file 2. [file 12863_2021_1018_MOESM2_ESM.tiff]

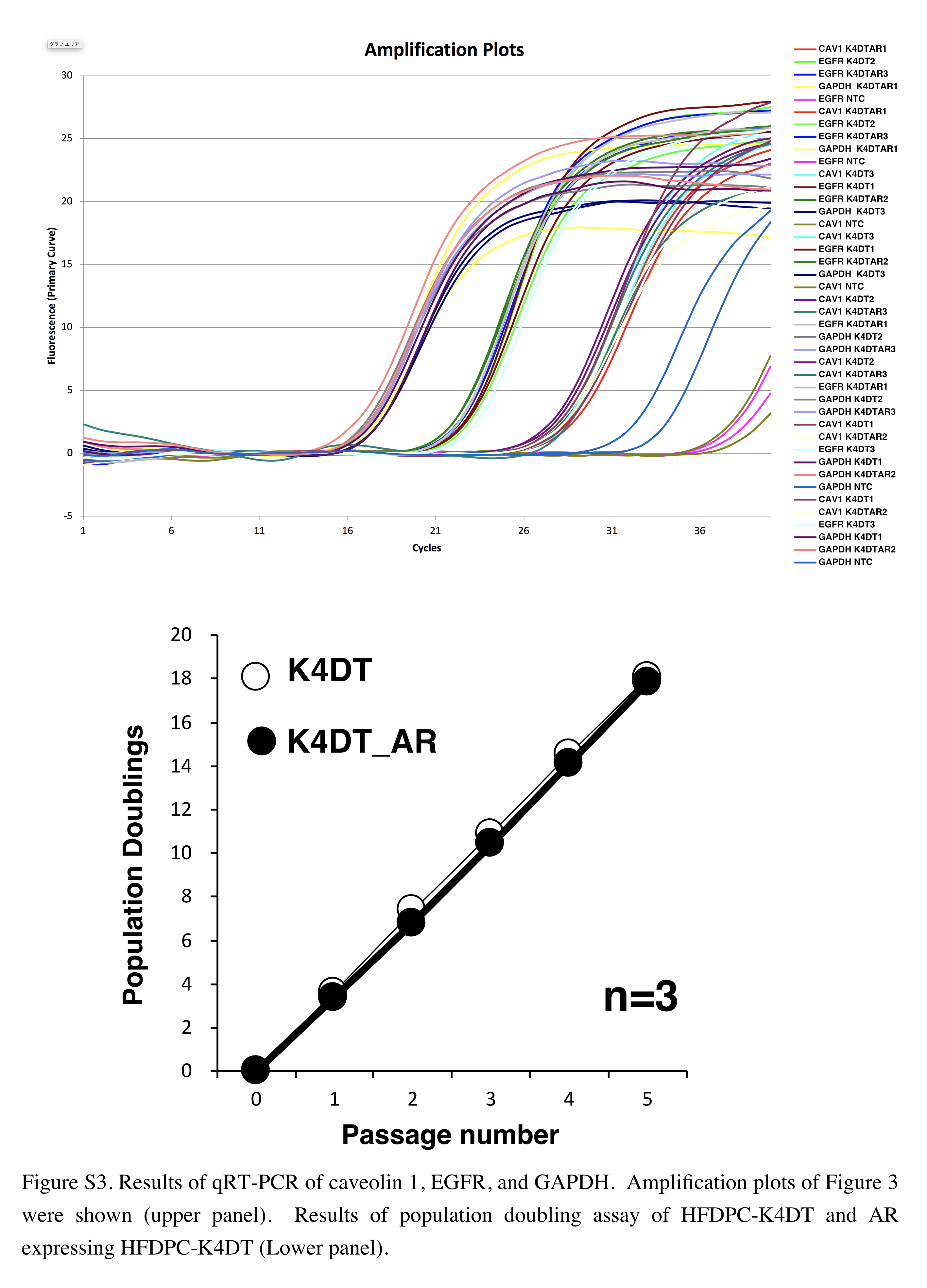

Supplement: Supplementary file 3 — Additional file 3. [file 12863_2021_1018_MOESM3_ESM.tiff]

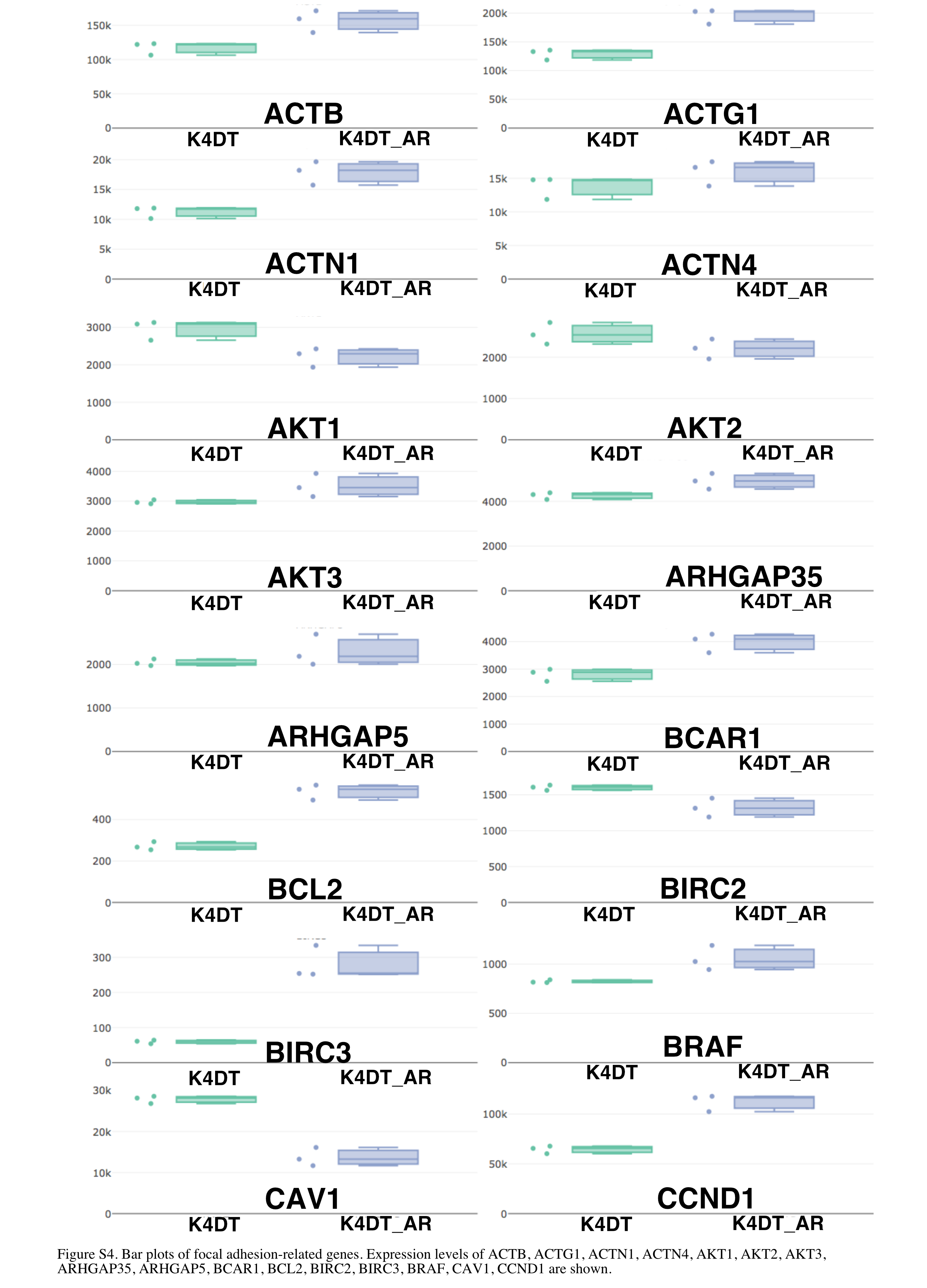

Supplement: Supplementary file 4 — Additional file 4. [file 12863_2021_1018_MOESM4_ESM.tiff]

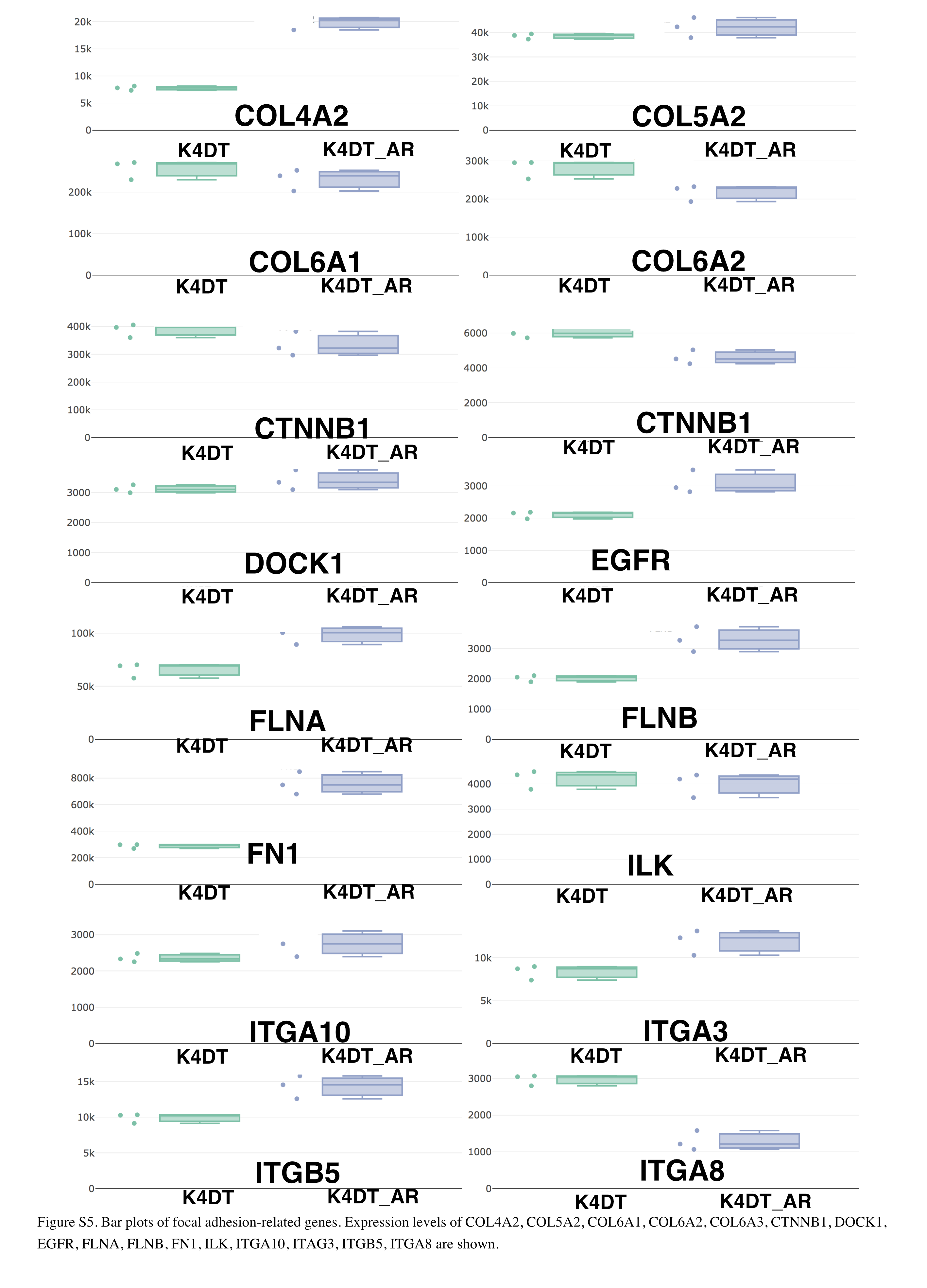

Supplement: Supplementary file 5 — Additional file 5. [file 12863_2021_1018_MOESM5_ESM.tiff]

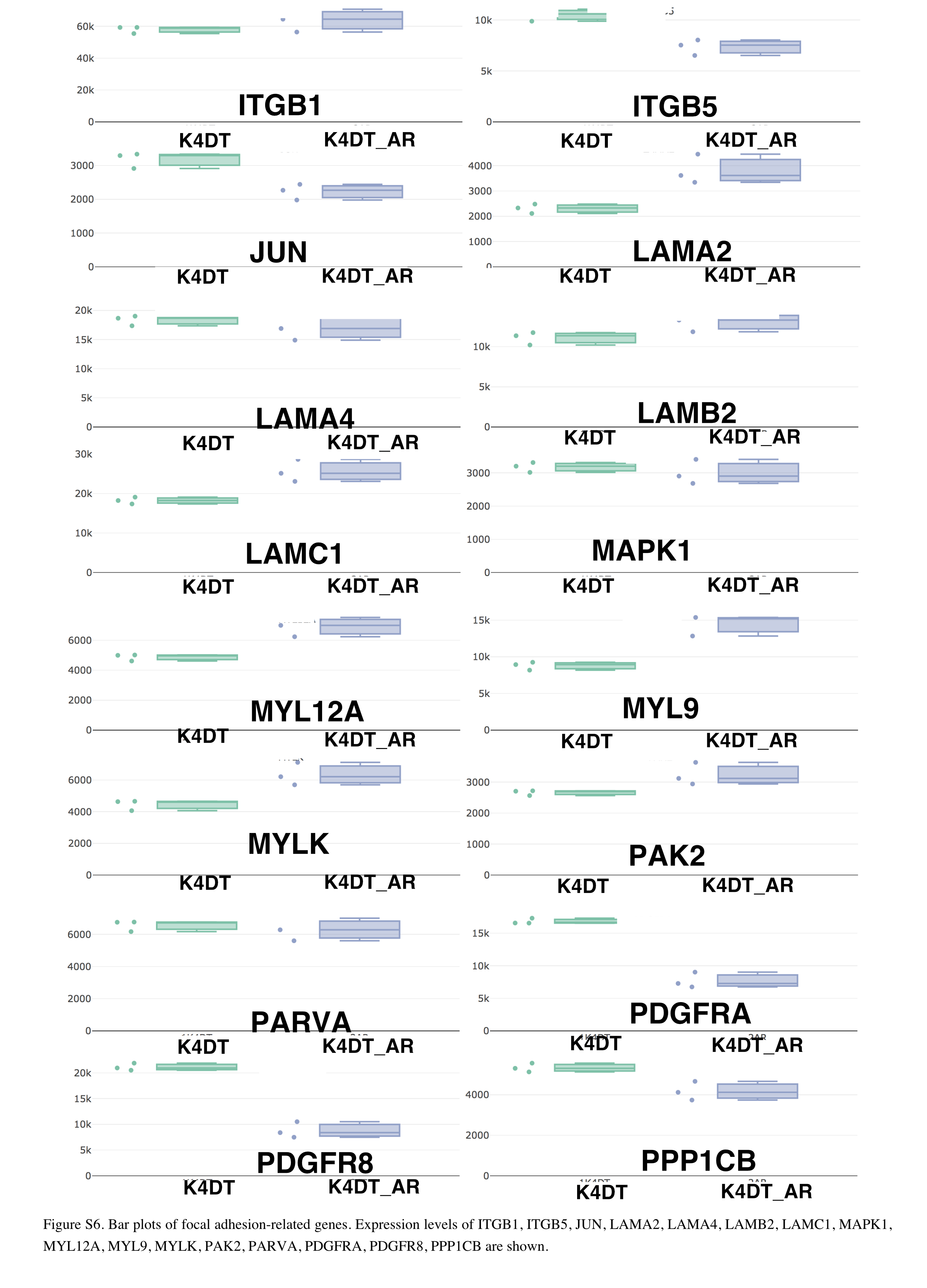

Supplement: Supplementary file 6 — Additional file 6. [file 12863_2021_1018_MOESM6_ESM.tiff]

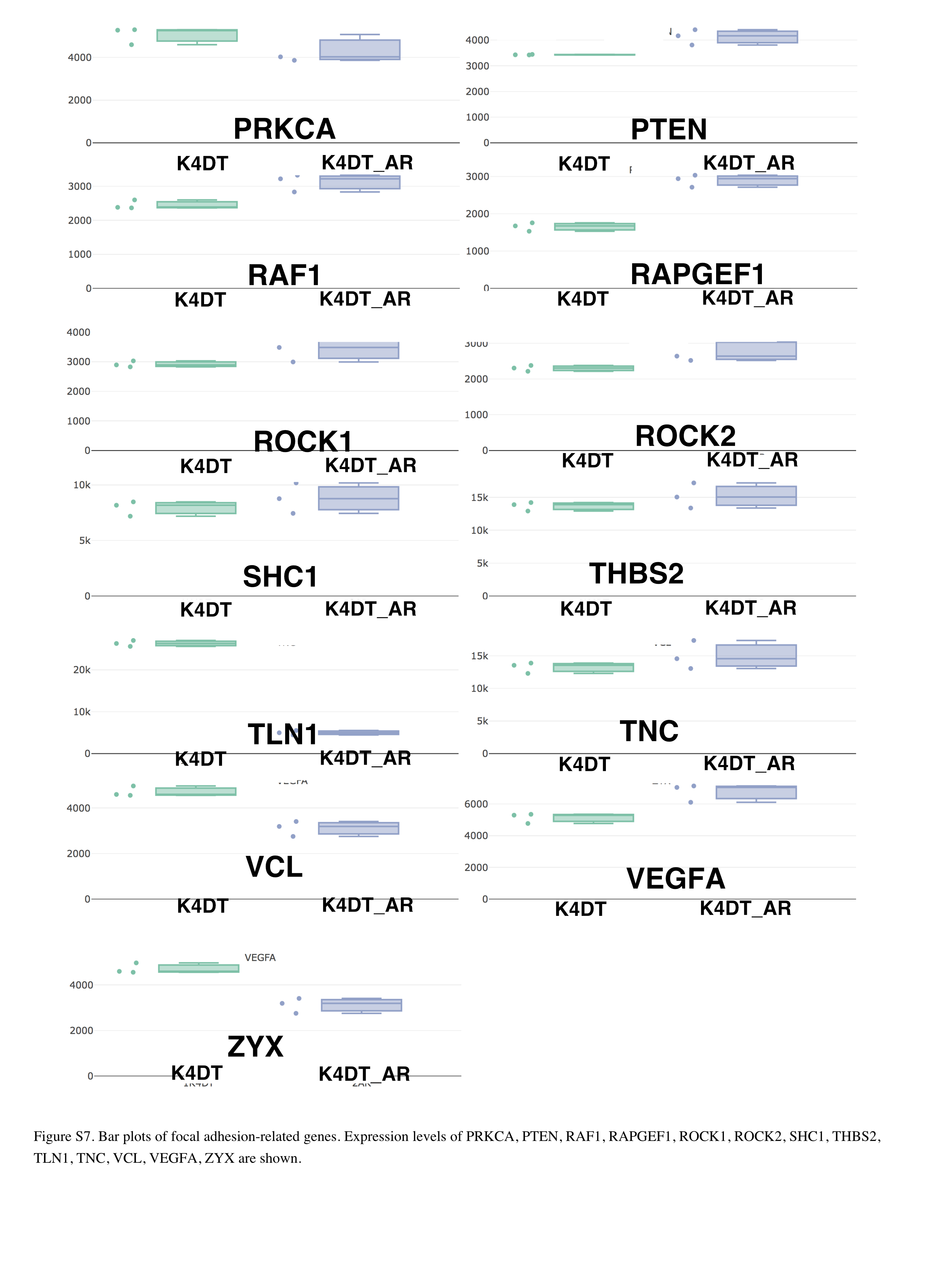

Supplement: Supplementary file 7 — Additional file 7. [file 12863_2021_1018_MOESM7_ESM.tiff]

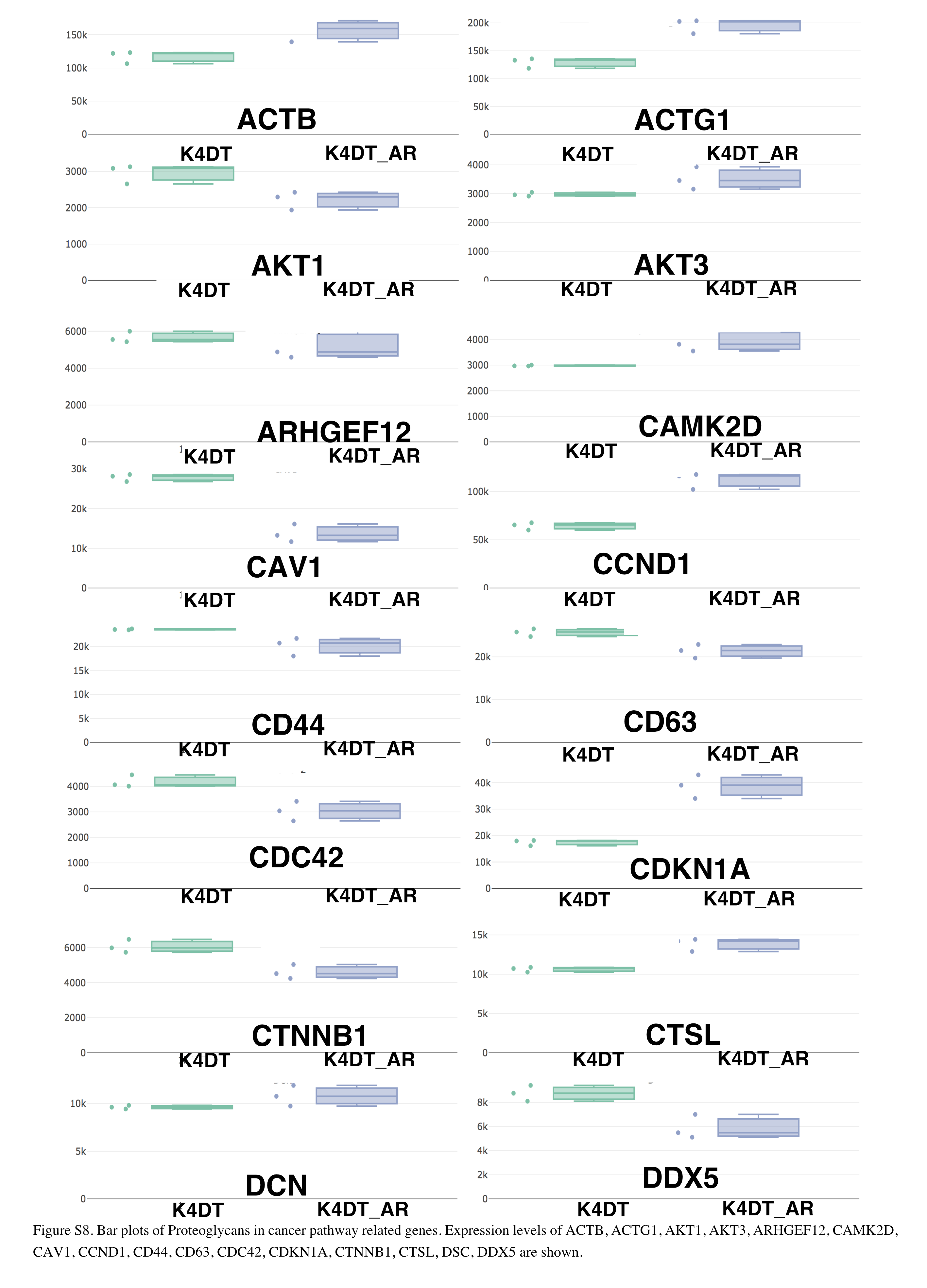

Supplement: Supplementary file 8 — Additional file 8. [file 12863_2021_1018_MOESM8_ESM.tiff]

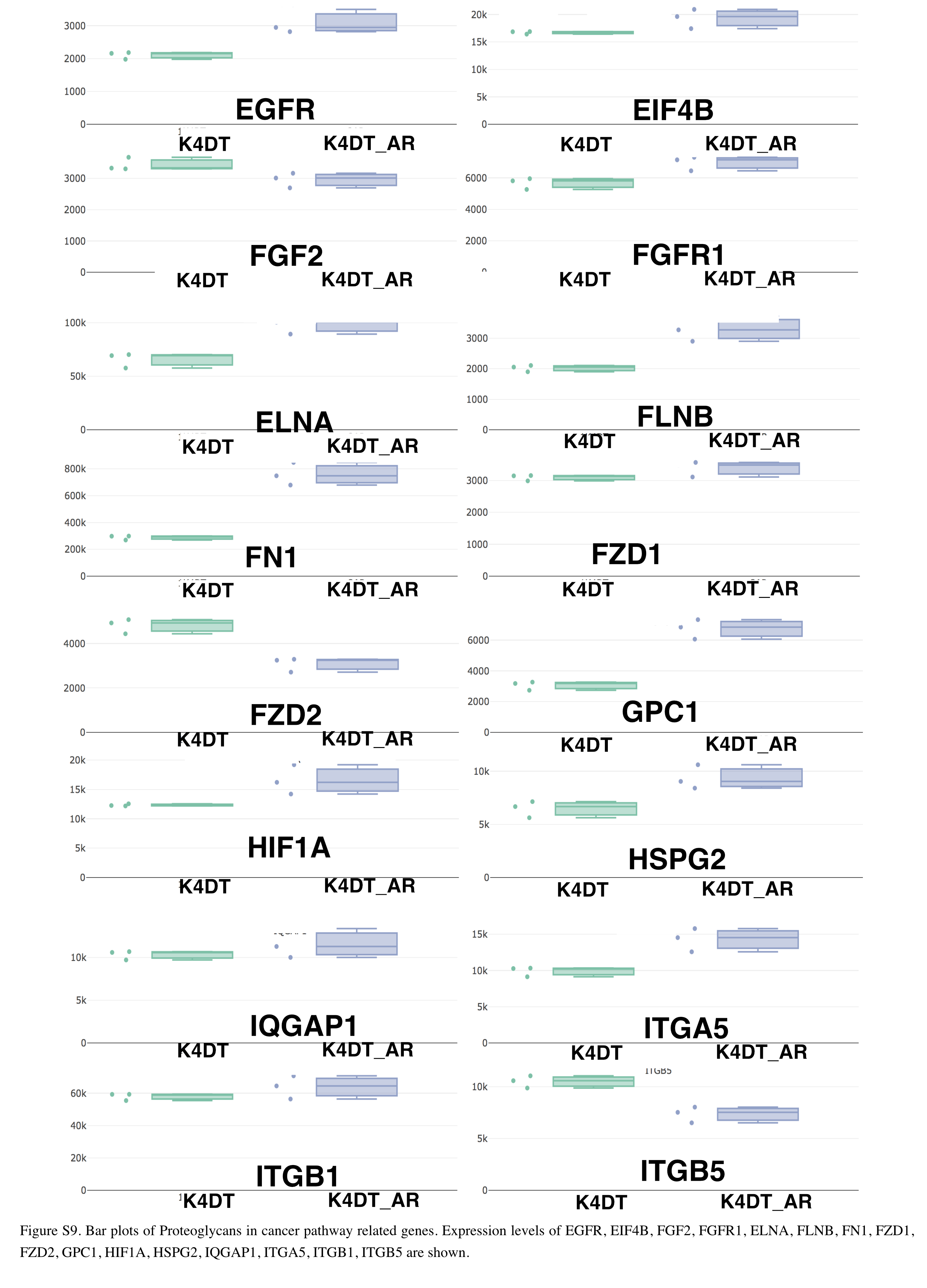

Supplement: Supplementary file 9 — Additional file 9. [file 12863_2021_1018_MOESM9_ESM.tiff]

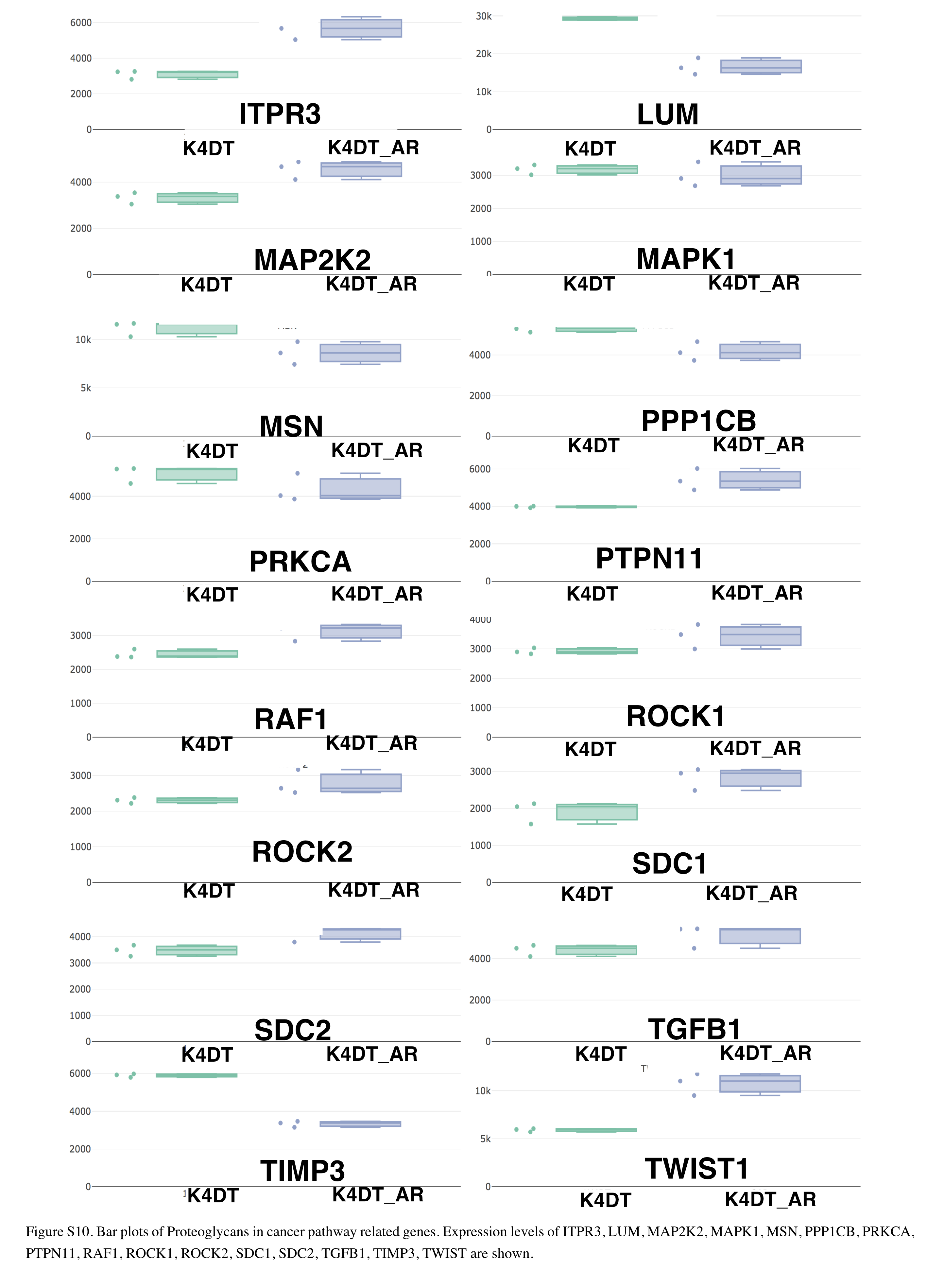

Supplement: Supplementary file 10 — Additional file 10. [file 12863_2021_1018_MOESM10_ESM.tiff]

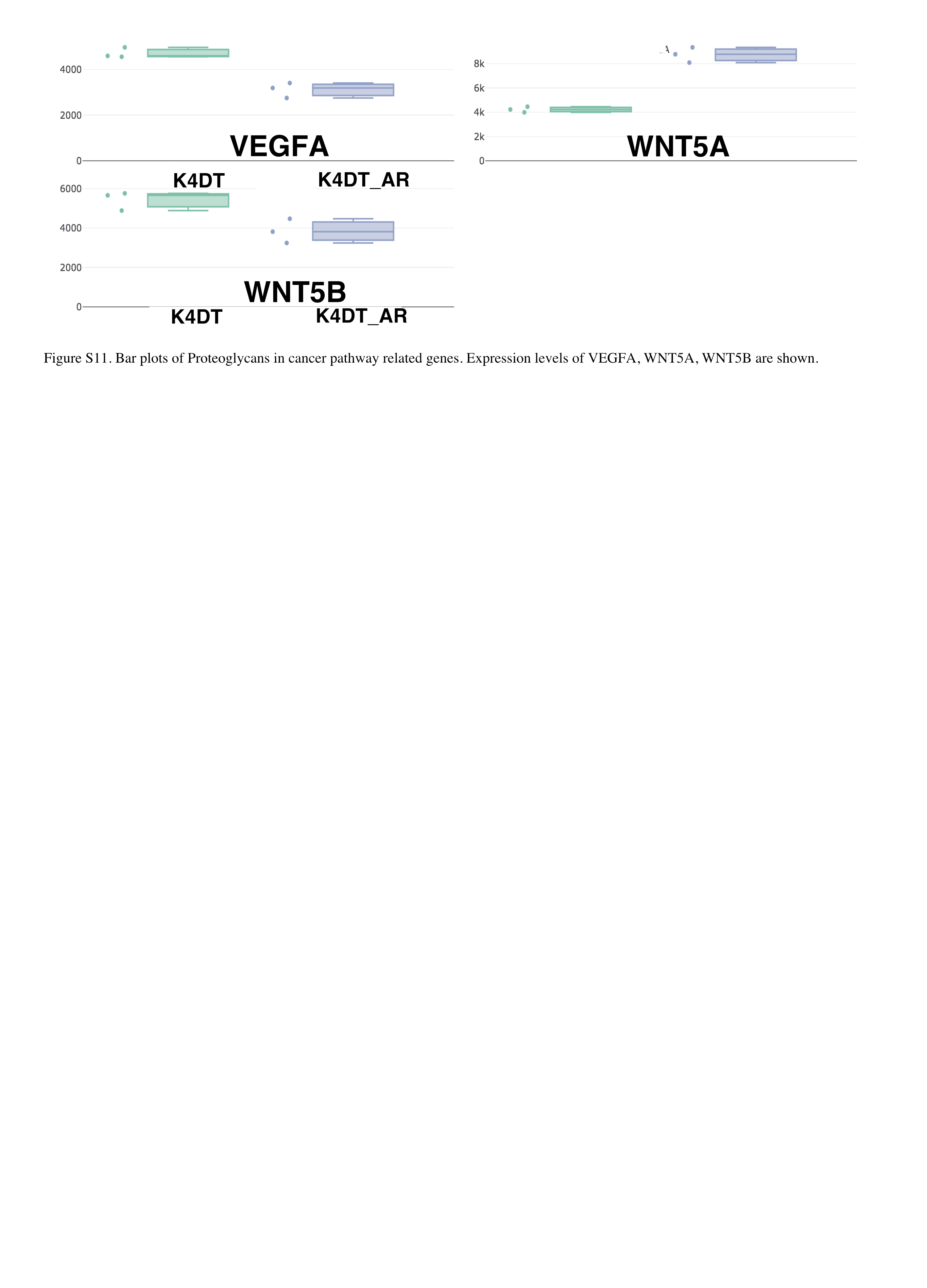

Supplement: Supplementary file 11 — Additional file 11. [file 12863_2021_1018_MOESM11_ESM.tiff]
